# Locally transmitted malaria in Tawau, Sabah, Malaysia

**Published:** 2017-10-01

**Authors:** Vivek J. Jayaraj, Dhesi Baharaja, Navindran Gopalakrishnan, Yomain Kaco

**Affiliations:** 1 Centre for Disease Control, Tawau District Health Office 91008, Tawau, Sabah, Malaysia; 2 Institute of Medical Research, Ministry of Health, Kuala Lumpur, Malaysia

## Abstract

**Background:**

Tawau was the epicentre of malaria infections in the 1970-1990’s, when industrialisation swept across the state of Sabah, Malaysia. Since then, effective public health intervention, mainly the Malaria Elimination Programme, introduced in 1998, has seen the disease shrivel down into its final elimination phase. Here we retrospectively analyse the case of a 63 year old male with multiple comorbidities who had no exposure to localities with high risk of infection- thus raising the question regarding the means of transmission.

**Materials and methods:**

Multiple interviews and an entomological survey were conducted to elucidate the possible mechanism of infection in this patient.

**Results:**

Findings point to locally-transmitted malaria, likely introduced by a patient from an endemic region in Tawau. Transmission via this route is rare, and has never before been reported in our setting.

**Conclusions:**

This rare case highlights the need for constant vigilance in malaria control and elimination, especially when the target of country-wide elimination is close.

## 1 Introduction

Autochthonous malaria is defined as a malaria infection that is acquired locally following the import of an infected vector from an endemic area or transmission by local mosquitoes following the introduction of an infected person [1-4]. *Plasmodium falciparum* has a relatively short incubation period and is unique in its virulence in that it indiscriminately infects erythrocytes of all ages [5]. The most important vector of malaria in Sabah is *Anopheles balabascensis,* a mosquito capable flying up to 2 km and breeding in small, temporary water bodies found mostly in dense tropical forest [6-8].

## 2 Materials and methods

### 2.1 Study site and population

Tawau is a district on the east coast of Sabah. It is made up of 6125 km^2^ of diverse geography ranging from urban city centres with outlying suburban districts, fishing and farming villages, palm oil plantations, logging sites and dense forest. The district has a tropical rainforest climate with average temperatures ranging from 26-29^0^C throughout the year. Generally, November, December and January experience the highest rainfall and February and March are the driest months. Malaria transmission is observed all year round. Since the induction of the Malaria Elimination Programme in 1998, the number of malaria cases has drastically reduced. Tawau itself is on the verge of malaria elimination – recording a mere 56 cases in 2016 compared to the thousands observed in the 1990’s. This has been achieved through a system of compartmentalising districts and stratification based on incidence and risk factors, rigorous health education and disciplined local application on indoor residual spraying (IRS) and insecticide-treated bednets. This achievement has resulted in the complete elimination of malaria in the urban and suburban city centre. All recent cases have been recorded in outlying districts, always near dense forest reserves or oil palm plantations. Over the last 15 years, naturally occurring malaria has not been observed in the urban city centre – the locality at which the case in question resides.

### 2.2 Diagnosis and treatment

The patient in question, his wife and son were interviewed during the examination. Clinical diagnosis was made by physicians in Tawau General Hospital, Sabah. Treatment was also guided by them. All clinical information was retrieved via case notes made available by the hospital and also through direct contact with the attending medical officers and physicians.

### 2.3 Entomological survey

A human landing catch was carried out by trained assistant environmental health officers from Tawau Health Office over a period of 3 days in sites at risk of harbouring vectors within a 2 km radius of both the hospital and the patient’s home. This was carried out in 2 shifts beginning at 6 pm to 8 am from 20-22 August 2016. A thorough check of the patient’s housing was also conducted.

## 3 Case report

The case occurred during the 2^nd^ week of August 2016. A 63-year-old male was suffering from multiple comorbidities at the time of admission. In 2008, he had suffered from a myocardial infarction (MI). He underwent a coronary angiography and was found to have a 2-vessel block. In the years following his MI, the patient developed congestive cardiac failure. He was classified using the New York Heart Association Classification (NYHA) as a Class III. He had also been admitted in 2008 for an upper gastrointestinal bleed. He was transfused with 2 units of fresh frozen plasma at that point. A subsequent endoscopy uncovered a Forest 3 ulcer of his stomach. He was put on proton pump inhibitors and discharged for follow up under the specialist clinics. He also suffered from Type 2 Diabetes, chronic kidney disease Stage 5, open angle glaucoma, benign prostate hyperplasia, and bilateral inguinal hernia. He was under multiple specialist clinic follow up for his comorbidities and was on multiple medications including Aspirin, Finasteride, Terazocin, Simvastatin and Glicazide. Due to his multiple problems, he was judged unfit for surgery and as such simply lived with his many problems. Although he was still able to carry out activities of daily life, he otherwise could not work for extended periods and found travelling extremely difficult. As such, he moved into a small room in the centre of town to ease the means of travelling about.

Prior to his admission for malaria, the patient was admitted for cholera between the 10-15 July. He had presented prior to the 10^th^ with a 3-day history of voluminous diarrhoea and stomach cramps. Upon testing in the emergency department, he tested positive on a stool culture for *Vibrio cholerae.* He was treated with antibiotics and fluids, and discharged after testing negative on 3 consecutive samples post treatment. On the 16^th^ of July, upon discharge, the patient complained of feeling unwell when he arrived home. In the proceeding 2 weeks he continued to feel unwell; complaining of fever with chills and rigours, malaise and loss of appetite. He was brought to the emergency department again on 1^st^ August 2016, in extremis. He had a blood pressure of 100/55 which dropped to 80/55 with a pulse of 160. He had a spike in temperature and was tachypnoeic. He was scored a GCS 14 initially, but consciousness level floated down to GCS 8 at one point. He was intubated for airway protection and was transferred to the ICU for intensive care and ventilation. In ICU, he was found to be anaemic. He also suffered from an acute kidney injury and metabolic acidosis. He was investigated for causes of acute pyrexia, and found to be positive for *Plasmodium falciparum* on a blood film stained with Giemsa 6% for identification of malarial parasites. Parasite count on the 1^st^ of August was 61511 parasites/μl. He was started immediately on intravenous artesunate on the 1^st^ of August. Response to treatment was immediate. There was improvement in all clinical and laboratory parameters. Blood film on day 2 still exhibited 88700 parasites/μl. On day 3, patient continued to improve, and parasitaemia had dropped to 2900 parasites/μl. All subsequent blood films were negative. He was extubated on day 3 in intensive care, and transferred to a general ward for further care. He was switched from intravenous artesunate to oral artemisinin-lumefantrine combination therapy on day 5 for a further 5 days. He received no transfusion of blood products during this hospitalisation or the one prior to this. He was discharged 10 days post admission. PCR for malaria confirmed diagnosis as *P. falciparum* 2 weeks post discharge and all cultures were sterile.

The means of infection with malaria in this patient is intriguing. Preceding his first admission during this period, dating 1 month prior to 10 July, the patient hardly moved. He frequented two establishments; for food and drink during this time which was a mere 100 m from his home in the centre of town. He denied travelling anywhere other than these two places. This was further verified by both his wife and son. His heart failure meant travelling anywhere more than a few metres was extremely challenging. The patient also did not own any transportation of his own, fully relying on the rather poor Tawau public transport service. During this period, a locality in Tawau known as Sg. Menteri was undergoing a *P. falciparum* epidemic. It had begun on 2 June 2016, and was propagated by hunters incompliant to their prophylaxis. Cases from this district were reported and even hospitalised, in close temporal proximity to our patient (Table 1). These patients had been admitted to the ward for antimalarial treatment. Two possibilities existed at this point – either there were competent anopheline mosquitoes in the immediate vicinity of the hospital that were capable of transmitting the parasite from the malaria patients in the ward or these patient from the Sg. Menteri epidemic who were admitted during that particular time period had brought in at least one infectious mosquito which then proceeded to feed on our patient.

**Table 1:** All *P. falciparum* cases occurring during Sg. Menteri Outbreak 2016. As can be seen there is an overlap during the patient’s stay in hospital for cholera and another patient (no. 9) with falciparum malaria.

Case no.	Age	Sex	Date of notification
1	41	Male	02/06/2016
2	40	Female	02/06/2016
3	25	Male	02/06/2016
4	42	Male	03/06/2016
5	38	Male	16/06/2016
6	15	Male	16/06/2016
7	23	Female	16/06/2016
8	46	Male	20/06/2016
***9***	***29***	***Male***	***11/07/2016***
10	34	Male	19/08/2016

On further history, the nature of this individual’s infection becomes even more complex. This gentleman had begun to work at the North Borneo Timber Company at Sebatik Island at the age of 23. He claimed to have worked there for slightly more than 20 years. He worked as a labourer mostly involved in the felling and transportation of trees. During his 20-year service there, during the late 1980’s or early 90’s, he claimed to have had malaria. He received treatment at the hospital and returned to work at the timber factory. He cannot remember if there were subsequent episodes of illness associated with malaria. He otherwise has no documentation from that time period suggesting that the diagnosis during that time was malaria, what form of malaria it was and what he was treated with. Nevertheless, it is known that during that period Pulau Sebatik was endemic for malaria, and the likelihood of a patient involved in the timber industry from that time period to contract malaria was very high. There is very little evidence though that can be sourced, from that time period regarding the epidemiology of malaria in Tawau during the late 80’s to the early 90’s. In the mid 1990’s, North Borneo Timber closed its operations in Sebatik Island. Thereafter, the patient in question took up many different jobs, mostly as a lorry driver in the palm oil industry. His job took him all over the east coast of Sabah – Kunak, Semporna and Tawau. He eventually retired at the age of 54 due to his illness. He receives welfare support from the government and financial assistance from both wife and son for his daily needs.

## 4 Discussion

As was described earlier, the patient had a previous infection of malaria close to 30 or more years ago. More recently, he developed a whole suite of comorbidities, including a very recent infection of *V. cholerae*. There is very little to suggest that any or all his comorbidities played a role in the pathogenesis of this falciparum malaria infection. Nevertheless, it is indeed interesting to note, that *V. cholerae* and *Plasmodium* have been suggested to have a link. More precisely, malaria seems to have a positively synergistic effect on a natural infection with cholera [9]. Would the inverse of this be possible and could it- the cholera infection, induce a malarial infection or relapse? There is no evidence currently to suggest this, yet it remains an interesting conundrum for further study.

As we know, *P. falciparum*, the parasite in question has only two means of recurrence [10]. These mechanisms are recrudescence or reinfection. Recrudescence can be due to resistance or improper use of antimalarials, antigenic variation and infection by different strains [10-13]. Recrudescence is generally thought to occur anywhere between 7 to 28 days after treatment but has been seen it particularly long latencies of up to 1 year [10,14-16]. Infections after 1 year are unlikely to be recrudescent in nature. In areas of high transmission there is no way to differentiate between recrudescence and reinfection. Hence the question whether or not our patient could have been suffering from a recrudescent infection. There is little to suggest this. Firstly, the primary infection took place 30 years ago, which is far longer than the possible latency associated with *P. falciparum*. Secondly, there is a distinct lack of documentation from his primary infection. There is nothing to suggest his primary infection was also caused by *P. falciparum*. There is no information on the drugs he was treated with. Most importantly, we do not know if in subsequent years he developed further episodes of malaria. Hence if this was not recrudescent malaria, could this represent some form of chronic dormant infection? With so little in terms of a reliable history on his previous infection, there is little proof to suggest this. That being said, some literature does shed light on the possibility of a chronic infection or dormant states of *P falciparum*. A single solitary nidus of sequestered *P. falciparum* was found in the placenta of a woman after delivery in Kenya [17]. Another case of man with a background of sickle cell anaemia was diagnosed with falciparum malaria 4 years after exposure; it was postulated that the parasite could have sequestered into microcirculation and remained dormant there [18]. These rare cases, raise an important question in the pathogenesis of *P. falciparum*. Is there likelihood of chronic infections, and if so where do they reside, and in what form?

As such, the most important fact to establish would be one related to movement of the patient in the incubation period preceding illness. First consider that the locality of our patient’s residence has been malaria free for a good 25 years. The patient also had a long history of being ill, since 2008. It is highly likely therefore that his story regarding limited movement in the months preceding his malaria infection was reliable. Hence if he did not travel to an endemic area, only an infectious individual could have brought the parasite close enough for a local mosquito to have transmitted the parasite to him. It turns out such a fortuitous event occurred around the 11^th^ to the 15^th^ of July, when an individual diagnosed with falciparum malaria was admitted to the adjacent ward. A 3-day long entomological survey in a nearby site with potential to harbour anophelines yielded negative results, which disproved the possibility of transmission by a local vector. Considering also the likelihood of greater incidence if there had been local infected mosquitoes at that time, the probability of local vector transmission seems remote.

Hence, after much debate and discussion, it only appears likely that this individual during his stay in the hospital for a cholera infection, became infected with *P. falciparum*. This had likely occurred when an infected *Anopheles* mosquito was brought into the ward premises, by someone from an endemic region. The suspect individuals were relatives of the falciparum malaria case in the adjacent ward. Retrospective history confirms, that during the 4 days both these patients were admitted, family members of the malaria patient had indeed visited him twice. They had driven there and brought a bag of clothes for him. Thus, through a series of highly coincidental and unfortunate events, we cannot confirm but conclude that our patient came to be infected with the *P. falciparum* parasite via an introduced infectious vector.

## 5 Conclusion

The means through which this gentleman contracted malaria were somewhat unique, bizarre and unfortunate. While this mechanism of transmission remains rare and highly unlikely, the occurrence of autochthonous malaria in Tawau, Sabah should not be taken lightly. A concentrated multidisciplinary effort will be necessary if we are to completely eliminate malaria from our shores. More research into the possible chronicity of *P. falciparum* is necessary, as is the need for stricter, up to date and more rigorous treatment guidelines. This would be to prevent the dormancy that is so frequently seen in recrudescent malaria. Whilst maybe a tad too aggressive, disinfection guidelines should be introduced into the hospital setting – if anything to reduce the possibility of transmission intra- hospital. The introduction of locality based diagnosis of malaria seems to be made more concrete by this case since the likelihood of malaria in the urbanised setting is extremely low.
